# Reevaluating the Concept of Treating Experimental Tumors with a Mixed Bacterial Vaccine: Coley's Toxin

**DOI:** 10.1155/2012/230625

**Published:** 2012-11-11

**Authors:** C. Maletzki, U. Klier, W. Obst, B. Kreikemeyer, M. Linnebacher

**Affiliations:** ^1^Section of Molecular Oncology and Immunotherapy, Department of General, Vascular, Thoracic and Transplantation Surgery, Schillingallee 69, 18055 Rostock, Germany; ^2^Division of Gastroenterology, Department of Internal Medicine, University of Rostock, 18055 Rostock, Germany; ^3^Department of Medical Microbiology and Hospital Hygiene, Institute of Medical Microbiology, Virology and Hygiene, University of Rostock, 18055 Rostock, Germany

## Abstract

Several decades after Coley's initial work, we here systematically analyzed tumoricidal as well as immunostimulatory effects of the historical preparation Coley's Toxin (CT), a safe vaccine made of heat-inactivated *S. pyogenes* and *S. marcescens*. First, by performing *in vitro* analysis, established human pancreatic carcinoma cell lines responded with dose- and time-dependent growth inhibition. Effects were attributed to necrotic as well as apoptotic cell death as determined by increased Caspase 3/7 levels, raised numbers of cells with sub-G1-DNA, and induced p21^waf^ expression, indicative for cell cycle arrest. Besides, CT effectively stimulated human peripheral blood leukocytes (huPBL) from healthy volunteers. Quantitative gene expression analysis revealed upregulated mRNA levels of selected Toll-like receptors. Flow cytometric phenotyping of CT-stimulated huPBLs identified raised numbers of CD25^+^-activated leukocytes. *In vivo*, repetitive, local CT application was well tolerated by animals and induced considerable delay of Panc02 tumors. However, systemic treatment failed to affect tumor growth. Antitumoral effects following local therapy were primarily accompanied by stimulation of innate immune mechanisms. Data presented herein prove that the historical approach of using killed bacteria as active immunotherapeutic agents still holds promise, and further careful preclinical analyses may pave the way back into clinical applications.

## 1. Introduction

Chronic infection can lead to cancer. However, acute infection has beneficial effects and often contributes to complete eradication of even large tumor burden. In this regard, the use of microbial vaccines for immunotherapy is still being re-examined. This therapeutic concept is based on the early work of William Coley, an American surgeon in the nineteenth century, who reported infection-associated tumor regression over a century ago. Inspired by his findings, he injected his first patients with vital *Streptococcus pyogenes*, a gram-positive organism causing erysipelas. Coley observed tumor shrinkage, but also lethal systemic infections. Thus, he modified his treatment regimen and since 1893 he has used a mixture of heat inactivated *S. pyogenes *and *Serratia marcescens*. The inoculation of this bacterial vaccine, later known as “Coley's Toxin” (CT), marked the origin of modern immunotherapy and Coley is also referred to as “father of cancer immunotherapy” [1]. 

Discoveries of the last decades comprehensively deepened our functional understanding of the immune system. Coley himself believed that the effect of his bacterial mixture is based on release of toxins affecting tumor but sparing normal cells [2]. In fact, CT activates the innate as well as the adaptive immune system by binding Toll-like (TLR) and other pattern recognition receptors. With regard to the bacterial nature of CT, this mixture contains unmethylated CpG, lipoteichoic acid, and lipopolysaccharide (LPS), acting agonistic with several TLRs [3]. Engagement of TLRs induces an inflammatory cascade resulting in cytokine secretion and immune cell activation [4]. This proinflammatory milieu together with high fever breaks the tumor-induced immune tolerance and changes it to an antitumor immunity [5–7]. However, his original hypothesis resting on an immune reaction against a “toxin” present in the microbial material that cross-reacts with and destroys the tumor cells fells more and more into oblivion and up to now has only partly been re-examined [1].

In the 1960s and 1970s, commercial CT preparations were tested on small patient cohorts. In these experiments, results were variable—presumably because of relatively short treatment courses. Also, most of the patients were immunocompromised due to prior chemotherapy [8, 9]. Besides, plenty immune mediators relevant for the inflammatory process were used as single agents in cancer immunotherapy [10–12]. But most of them failed to prove clinical efficiency. Indeed, the benefit of CT treatment is supposed to base on the chronological sequence of single immune mediators to induce an optimal antitumor immune response. These facts strengthen the usefulness of a comprehensive analysis regarding the therapeutic potential of the toxin, generated from the original protocol. In 2005, a Canadian company (MBVax) started to produce CT and rekindle Coley's pioneer work. Since then, promising results for different tumor entities were obtained. These findings are an inducement for further investigations on the antitumoral efficiency of CT. Given the lack of experimental data for pancreatic carcinomas, we here picked up the idea of using CT for cancer immunotherapy. We comprehensively analyzed the potential of this toxin to affect tumor cell growth both *in vivo* and *in vitro*, taking advantage of its intrinsic immune stimulatory properties. We observed a direct impact on cell growth, proliferation, and viability. Of note, proapoptotic molecules in tumor target cells increased upon CT treatment. *In vivo*, repetitive local CT applications effectively controlled tumor growth by stimulating immune responses. 

## 2. Material and Methods

### 2.1. Tumor Cell Lines, Human Peripheral Blood Lymphocytes (huPBL), and Culture Media

Human (AsPC-1, T3M4, MIA PaCa-2, and BxPc-3) and murine (Panc02) pancreatic carcinoma cell lines were maintained in DMEM/HamsF12 supplemented with 10% fetal calf serum (FCS), glutamine (2 mmol/L), and antibiotics (complete medium). Human peripheral blood leukocytes (huPBL) were isolated from blood of healthy donors with no known diseases. Purification of huPBLs was performed by Ficoll density-gradient centrifugation and subsequent cultivation in IMDM supplemented with 10% fetal calf serum (FCS), glutamine (2 mmol/L), and antibiotics. All media and supplements were from PAA unless stated otherwise (Cölbe, Germany).

### 2.2. Preparation of Coley's Toxin

Coley's Toxin (CT) was prepared following the original protocol. Briefly, A single group A streptococcal isolate, *S. pyogenes* serotype M49 (strain 591), was cultured in Todd-Hewitt (TH) broth (Oxoid Unipath, Wesel, Germany), supplemented with 10% glucose, and allowed to grow for ten days in an incubator (37°C, 5% CO_2_). An aliquot was taken from the culture, plated on sheep blood agar, and incubated overnight to examine cell viability. Afterwards, the culture was seeded with 2 mL of live *S. marcescens* (2 ∗ 10^7^ cfu/mL) and further cultured at 25°C. Following a period of ten days, incubation was terminated by heat sterilization (65°C for two hours) and subsequent filtration. Bacterial inactivation was confirmed after plating on sheep blood agar and overnight incubation. Prior to cell treatment, CT was pelleted and diluted in sterile complete medium to a final concentration corresponding to 5 ∗ 10^2^, 2.5 ∗ 10^4^, and 2.5 ∗ 10^6^ cfu/mL. 

### 2.3. RNA Isolation, cDNA Synthesis, and Quantitative Real-Time PCR

Total RNA from treated and untreated tumor cells as well as from huPBLs was isolated with TRIzol reagent according to the manufacturer's instructions. RNA was reverse transcribed into cDNA from 0.5 *μ*g RNA using the High Capacity cDNA Reverse Transcription Kit (Applied Biosystems, Foster City, CA, USA). Target cDNA levels were analyzed by quantitative real-time PCR using TaqManTM Universal PCR Master Mix and predesigned TaqMan gene expression assays (Hs00180269_m1 (Bax), Hs00355782_m1 (p21^waf^), Hs01014511_m1 (TLR2), Hs00152939_m1 (TLR4), Hs00152825_m1 (TLR5), Hs00152973_m1 (TLR9), and Hs99999905_m1 (GAPDH, housekeeping gene control) in an ABI Prism 7000 sequence detection system (Applied Biosystems). PCR conditions were as follows: 95°C for 10 min, 40 cycles of 15 s at 95°C, 1 min at 60°C. Reactions were performed in triplicate wells and replicated three times. Expression levels of the gene of interest are given in relation to the housekeeping gene (ΔCt = Ct_target_ − Ct_GAPDH_). Relative gene expression values are expressed as *x*-fold increase compared to untreated (i.e., tumor cells or huPBL) cells, whose expression was set to be 1.

### 2.4. Quantification of Cell Proliferation by BrdU Incorporation

Quantification of DNA synthesis was performed by measurement of 5-bromo-2′deoxyuridine (BrdU) incorporation using BrdU labeling and detection enzyme-linked immunosorbent assay (ELISA) kit (Roche). BrdU labeling was initiated after 24 and 48 hours of incubation with or without CT by addition of labeling solution at a final concentration of 10 *μ*M. Following the incubation period of three hours, labeling was stopped and BrdU uptake was quantified on a plate reader at 450 nm according to the manufacturer's instructions. Percentage of proliferating cells was calculated compared to untreated control (=100%).

### 2.5. Cell Viability Staining

Cell viability was determined by fluorescent staining using Calcein AM (Sigma, final concentration: 2 *μ*M). Analysis was performed on a fluorescence multiwell plate reader (Tecan Infinite M200, Crailsheim, Germany) at an excitation wavelength of 485 nm (emission 535 nm). For estimation of cell viability, the relative fluorescence intensities of Calcein AM-stained nontreated cells (=live control) were set to be 100%, and fluorescence intensities of the samples were calculated. Experiments were performed in duplicates and replicated at least three times. 

### 2.6. Apoptosis Assays

Cells were grown to about 70% confluence in 24-well culture plates and then incubated in 2 mL complete medium with or without bacteria for 24 and 48 hours. At the end of treatment, the cells were harvested by trypisinzation, pelleted by centrifugation, washed twice with PBS and resuspended in 2 mL ice-cold 70% ethanol for at least 24 hours at −20°C. Afterwards, the cells were pelleted, washed with PBS, and incubated for one hour in 200 *μ*L PBS containing 0.1% Tween 20 and 1 mg/mL RNase (Sigma Aldrich, Munich, Germany) at room temperature. Following the addition of 50 *μ*g propidium iodide/10^6^ cells (Sigma), the samples were subjected to flow cytometric analysis which was performed on a FACSCalibur Cytometer (Becton Dickinson) using the Cellquest program. Ten thousand events were measured for each sample.

Additionally, apoptosis induction was examined via caspase activity. Cells were cultured in 96-well half-area plates and treated with increasing CT doses for 24 and 48 hours. Caspase 3/7 activity was quantified using the Promega ApoTox-Glo Triplex Assay according to the manufacturer's instructions (Promega, Mannheim, Germany) and measurement on a luminometer (Promega). Background luminescence (cell culture medium without cells and Caspase-Glo 3/7 Reagent) was subtracted from all measurements. Luminescence values of nontreated cells (negative control) were set to be one and *x*-fold increases in caspase activity of samples were calculated. Experiments were performed in duplicates and replicated at least three times. 

### 2.7. Flow Cytometric Phenotyping

HuPBLs were harvested for phenotyping following 24 hours of exposure towards increasing concentrations of CT. Therefore, huPBLs were stained with the following FITC- and PE-conjugated mouse antihuman monoclonal antibodies (mAbs): CD3, CD4, CD8, CD25, CD16, and CD56 (1 *μ*g, Immunotools, Friesoythe, Germany). Negative controls were stained with the appropriate isotypes (Immunotools). Additionally, fractions of dead cells were quantified after 24 hours, 72 hours, and 7 days using propidium iodide (PI). Therefore, PI (1 mg/mL) was given to cells directly prior to measuring. Phenotyping of murine immune cells was conducted from blood samples and spleens, following lysis of erythrocytes in lysis buffer (0.17 M Tris, 0.16 M NH_4_Cl) and labeling with the following FITC-conjugated rat antimouse mAbs: CD3, CD11b, CD19 (1 *μ*g, Immunotools), hamster antimouse mAbs: CD11c (1 *μ*g, Miltenyi Biotec, Bergisch-Gladbach, Germany) and PE-conjugated rat antimouse mAbs: CD4, CD8, Gr1 (Ly6G) (Miltenyi Biotec), and g/d TCR. Negative controls consisted of spleen and blood lymphocytes stained with the appropriate isotypes (BD Pharmingen). Samples were analyzed on a FACSCalibur Cytometer (BD Pharmingen). Data analysis was performed using CellQuest software (BD Pharmingen).

### 2.8. Co-Culture Experiments

Direct tumor cell killing in the presence of huPBLs was examined by co-culture experiments following 48 hours of incubation. Experiments were performed as described before [13]. Data are given as *x*-fold number of tumor cells compared to untreated controls. 

### 2.9. Pancreatic Tumor Model and Treatment Regimen

Experiments were performed on female 8–10-week-old C57Bl/6N mice (Charles River, Fa. Wiga, Sulzfeld, Germany) weighing 18–20 g. All animals were fed standard laboratory chow and given free access to water. Experiments were performed in accordance with the German legislation on protection of animals and the Guide for the Care and Use of Laboratory Animals (Institute of Laboratory Animal Resources, National Research Council; NIH Guide, vol. 25, no. 28, 1996). Under brief ether anesthesia 1 ∗ 10^6^ Panc02 cells were injected subcutaneously (s.c.) into the right hind leg. Tumor growth was routinely controlled at least twice a week and tumor volume was estimated according to the formula: *V*= width²  ∗ length ∗ 0.52. After tumor establishment animals were subdivided into the following experimental groups: animals received twice weekly intratumoral CT injections (i.t., resuspended in 50 *μ*L PBS), a total of six times (*n* = 5–7 per group). Another group was biweekly treated via tail vein injection (i.v., six injections in total, *n* = 3-4). As controls, one tumor-carrying group received PBS (vehicle) alone.

Tumor-carrying mice (treatment, control) were sacrificed at day 28 or when they became moribund before tumor volume reached 2000 mm³. At the end of each experiment, blood samples, tumor, spleen, and mesenteric lymph nodes were removed from the animals of all groups for further analysis.

### 2.10. Statistical Analysis

Values are reported as the mean ± SEM for *in vitro* and *in vivo* data. After proving the assumption of normality, differences were determined by using the unpaired Student's *t*-test. If normality failed, the nonparametric Mann-Whitney *U*-Test was applied. The tests were performed by using Sigma-Stat 3.0 (Jandel Corp, San Rafael, CA). The criterion for significance was set to *P* < 0.05.

## 3. Results

### 3.1. Inhibition of Tumor Cell Proliferation and Viability

**Figure 1:**
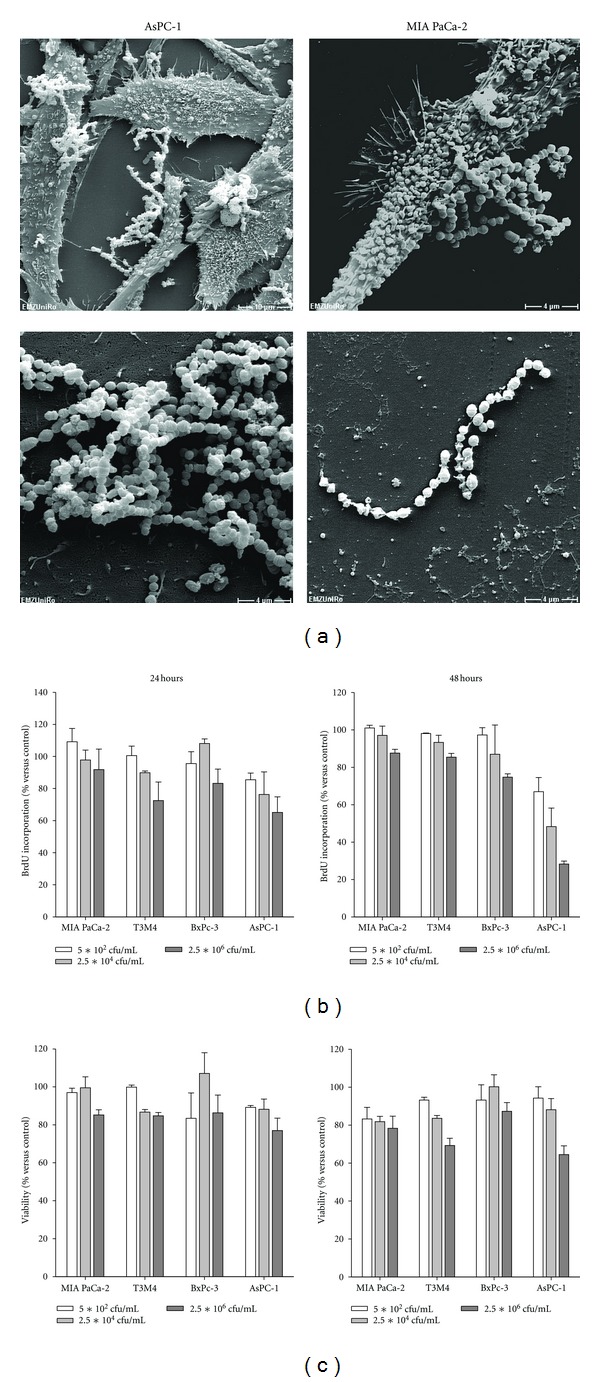
Electron microscopy. (a) Representative pictures showing CT binding to tumor cells via surface molecules (upper panel). Sustained cellular integrity despite heat inactivation was confirmed (lower panel). Cell proliferation and viability. (b) Tumor cells were seeded in 96-well plates and cultured with increasing CT concentrations (corresponding to 5 ∗ 10^2^, 2.5 ∗ 10^4^, and 2.5 ∗ 10^6^ cfu/mL) for 24 and 48 hours. Thereafter, cell proliferation was estimated by BrdU uptake and quantification on a plate reader at 450 nm according to the manufacturer's instructions. Percentage of proliferating cells was calculated compared to untreated control (100%). (c) For estimating viability, tumor cells were seeded in 24-well plates and treated with CT as described above. Following the incubation period (24 and 48 hours), the cells were stained with Calcein AM and fluorescence intensity was measured on a plate reader (ex/em 485/535 nm). Cell viability was calculated by setting relative fluorescence intensities of Calcein AM-stained nontreated cells (live control) to 100%. Results show data of three separate experiments each performed in duplicates. Values are given as the mean ± SEM.

To evaluate the direct impact of CT on cell proliferation and vitality, tumor cells were first treated with increasing bacterial doses (corresponding to 5 ∗ 10^2^, 2.5 ∗ 10^4^, and 2.5 ∗ 10^6^ cfu/mL). Electron microscopy following 24 hours of treatment demonstrated close adherence of CT to tumor cells via surface molecules, a characteristic common to *S. pyogenes* and *S. marcescens *(Figure 1(a), upper panel). Moreover, sustained structural integrity of bacteria was confirmed in these analyses (Figure 1(a), lower panel). 

Functional proliferation experiments displayed a time and dose-dependent tumor cell growth inhibition (Figure 1(b)). As determined by BrdU incorporation, most pronounced effects were observed for AsPC-1 cells. Cell growth significantly decreased to 80% and 28% after 24 and 48 hours, respectively, (compared to untreated controls). T3M4, BxPc-3, and MIA PaCa-2 cells were less susceptible towards CT. Here, growth inhibition was rather transiently with a maximum of 28% observed after 24 hours in T3M4 cells (highest dose). 

Antiproliferative effects could partly be attributed to cytotoxicity. Numbers of viable tumor cells were diminished, especially in the highest dose following a 48-hour incubation period (Figure 1(c)). Again, CT-mediated killing was most evident in AsPC-1 cells. Viability decreased time and dose dependently. Comparable, though less pronounced, effects were obtained for T3M4 and MIA PaCa-2 cells with a maximum of 20% dead cells (compared to untreated controls). Viability of BxPC-3 was only marginally affected by CT.

### 3.2. Induction of Cell Cycle Regulators

**Figure 2:**
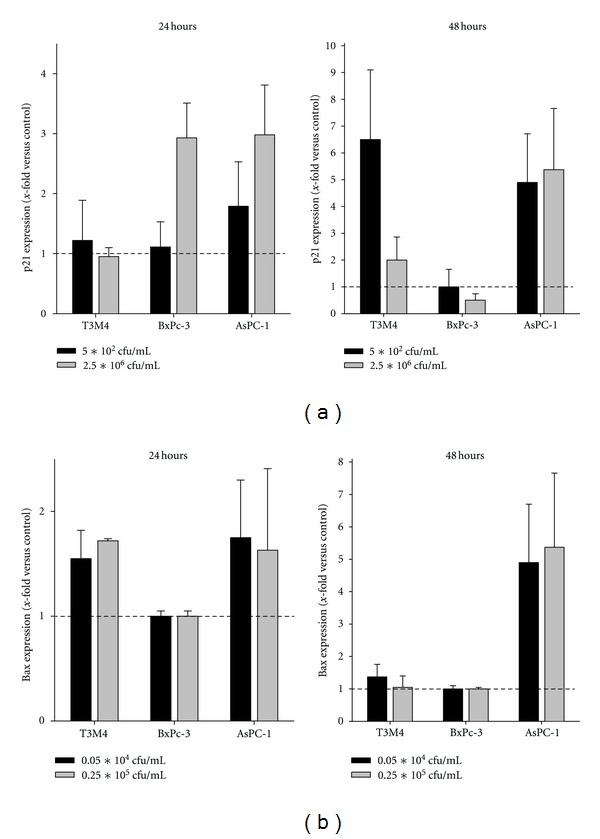
Gene expression analysis. mRNA expression of (a) p21^Waf^ and (b) Bax in tumor target cells following 24 and 48 hours CT treatment. Quantitative real-time PCR was performed using predesigned TaqMan gene expression assays. Results show data of three separate experiments each performed in duplicates. Values are given as the mean ± SEM.

As a first step towards functionally understanding the growth inhibitory and cytotoxic capacity of CT on tumor cells, gene expression of the cell cycle and DNA replication regulator p21^waf^ as well as apoptotic activator Bax was examined. Due to technical limitations, gene expression could only be considered for three out of the four cell lines studied. Analysis revealed increased expression levels of both target genes (Figures 2(a) and 2(b)). In detail, p21^waf^ expression was transiently induced in BxPC-3 target cells. T3M4 cells displayed a delayed response with highest expression levels after 48 hours. In those cells, expression correlated inversely with CT doses. AsPC-1 cells, whose viability was most affected by CT, reacted with sustained high p21^waf^ expression. The effects of CT on Bax gene expression were comparably mild, with a fivefold upregulation in AsPC-1 as the most pronounced phenomenon. As can be depicted from Figure 2(b) (lower panel), expression was only slightly stimulated in the other cell lines.

### 3.3. Apoptosis Induction following CT Exposure

**Figure 3:**
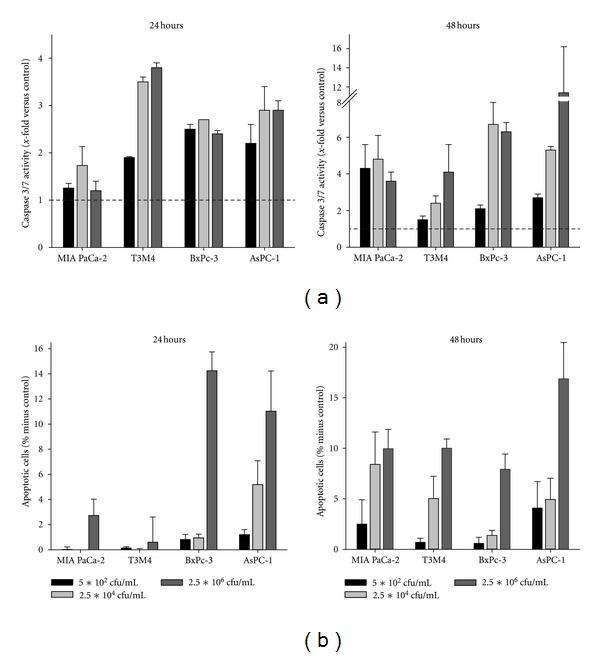
Apoptosis assay. (a) Increased Caspase 3/7 activity in tumor cells after CT treatment, as quantified using the Promega Caspase-Glo 3/7 Assay according to the manufacturer's instructions and measurement on a luminometer. (b) Flow cytometric sub-G1 Peak analysis following 24 and 48 hours of incubation with CT. Cells with DNA content lower than the G1 peak were considered to be apoptotic. Results show data of three separate experiments each performed in duplicates. Values are given as the mean ± SEM. (c) AsPC-1 cells show characteristic sub-G1 peaks at 24 hours after treatment (representative results out of three independent experiments).

Aforementioned analysis hinted towards potential contribution of CT-induced apoptosis induction in tumor target cells. Consequently, caspase activity as well as DNA fragmentation were investigated next. Caspase 3/7 levels dose and time dependently increased in all cell lines exposed to CT (Figure 3(a)). Levels showed up to sevenfold increases in BxPC-3 cells after 48 hours of treatment with intermediate and highest CT doses. Supplemental to the prior observation on highest susceptibility of AsPC-1 cells towards CT, maximal caspase activation was found in these cells after 48 hours. Likewise, DNA fragmentation, as determined by flow cytometric sub-G1-peak analysis, was most evident here (Figure 3(b)). In MIA PaCa-2, T3M4, and BxPC-3 cells, the fraction of apoptotic tumor cells also increased dose dependently (Figure 3(b)). 

Further determinations on cell cycle analysis revealed rearrangement of cell phases. In line with increased p21^waf^ expression, cell cycle arrest and accordingly loss of G2/M phase were apparent in CT exposed cells (Table 1). Therefore, mechanisms independent from apoptosis are also likely to be involved in the exerted cytotoxic reactivity. 

### 3.4. CT Activates Immune Cells in a Dose-Dependent Manner

Next, CTs' potential to stimulate huPBLs was examined. Given that CT is made of two bacterial species, that is, the gram-negative *S. marcescens* and the gram-positive *S. pyogenes*, we first determined mRNA levels of TLRs, known to be activated by microbial ligands.

**Figure 4:**
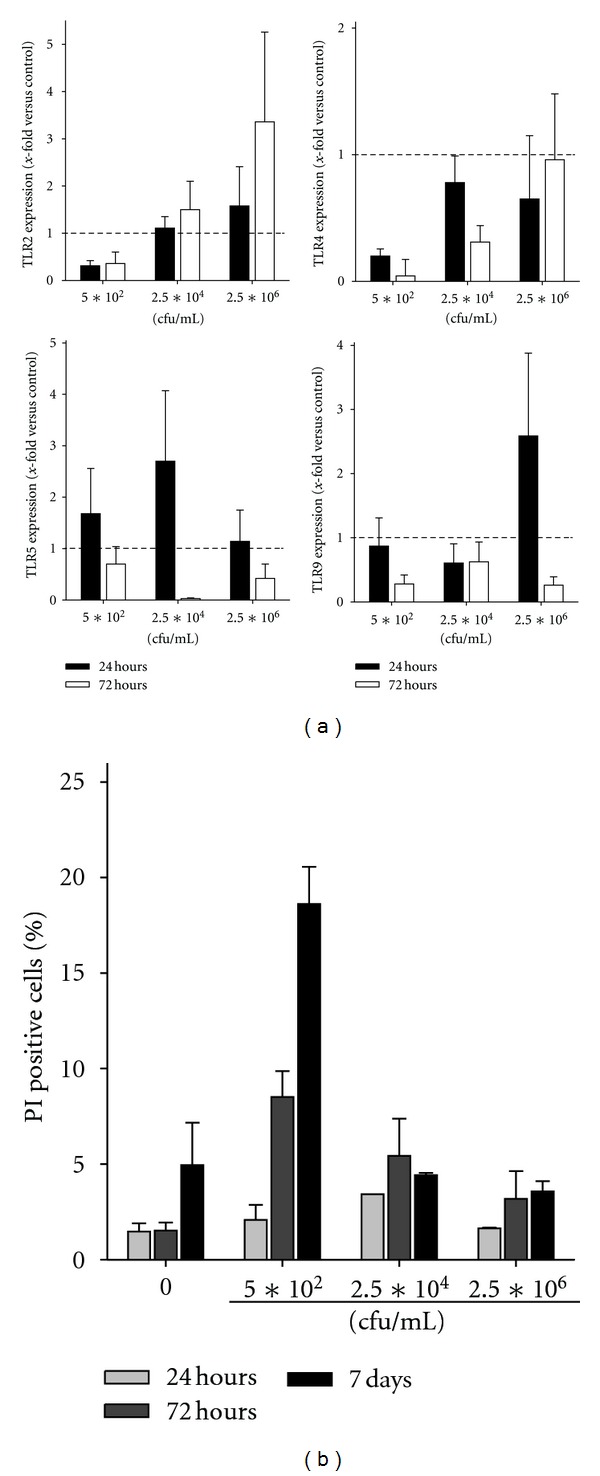
Gene expression analysis and viability of huPBLs. mRNA expression of (a) TLRs in huPBLs following 24 and 72 hours stimulation with CT. Quantitative real-time PCR was performed using predesigned TaqMan gene expression assays. Results show data of three separate experiments each performed in duplicates. (b) Viability of huPBLs as determined by flow cytometric PI staining. Prior to analysis, huPBLs were stimulated with increasing doses of CT and cultured for 24 hours, 72 hours, and 7 days. Results show data from four different healthy donors. Values are given as the mean ± SEM.

These analyses showed a dose- and time-dependent increase in TLR expression (Figure 4(a)). TLR2 displayed a maximal response after 72 hours stimulation with the highest CT concentration. Conversely, TLR5, the receptor for flagellin, and TLR9, recognizing unmethylated CpG islands of bacterial DNA, were transiently stimulated with up to threefold increases following 24 hours. The effects of CT on TLR4 could be neglected, since there was no stimulation at any time.

Further flow cytometric analysis revealed a dose-dependent rise in activated immune cells after 24 and 72 hours. Numbers of CD25^+^ cells increased more than threefold (Table 2). Further analysis identified CD3^+^CD8^+^ cytotoxic T cells to be the main responding cell population. Fractions of CD3^+^CD4^+^ T cells as well as CD16^+^CD56^+^ NK cells were only marginally influenced by CT. 

Moreover, CT mediated no significant cytotoxicity towards huPBLs. Numbers of PI^+^ cells at 24 and 72 hours of culture were always below 10% and thus comparable to nonstimulated huPBLs (Figure 4(b)). Interestingly, there was an inverse correlation between toxicity and CT dose. Highest PI^+^ levels were observed for lowest CT concentrations following 7 days of culture (Figure 4(b)). This might be best interpreted as a kind of missing stimuli at these low concentrations, resulting in leukocyte cell death. 

### 3.5. Boosted Antitumoral Effect by Adding huPBLs to the Culture

**Figure 5:**
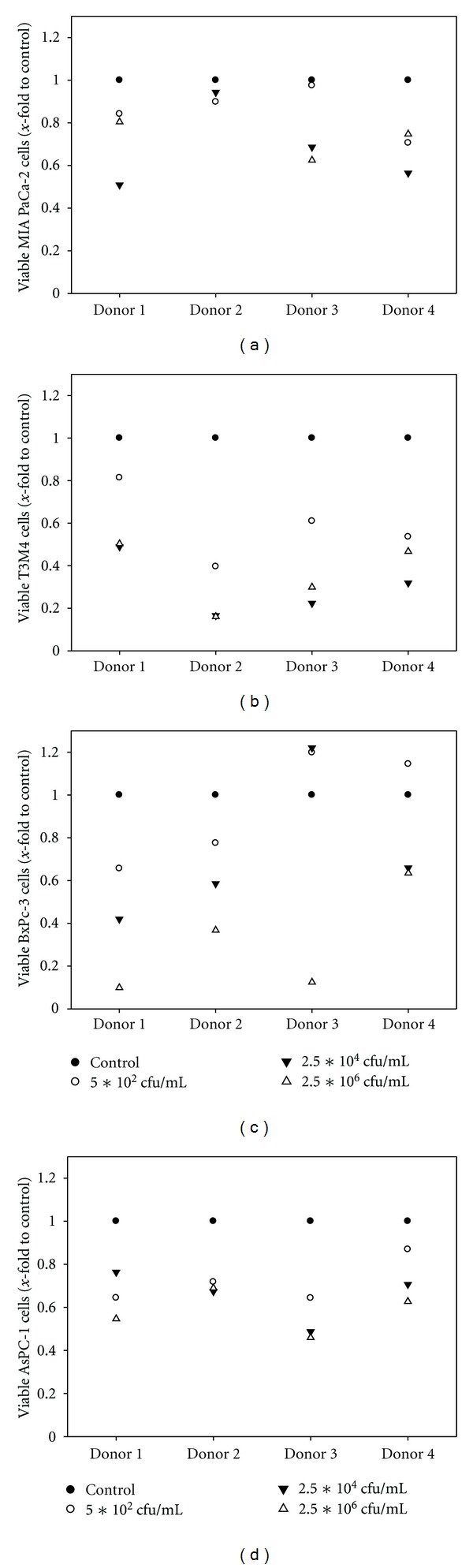
Co-culture experiments. Tumor cells were Co-cultured with huPBLs of four different healthy donors in the presence of CT for 48 hours. Thereafter, numbers of viable tumor cells were quantified by flow cytometry using microsphere beads as calibrator. Untreated cells without CT were set as 1 and all other data were given as *x*-fold increase. Experiments were performed in duplicates.

Based on the observation that CT acts as immunostimulatory and antitumoral agent, a series of *in vitro *co-culture experiments was performed by adding immunocompetent cells and CT to tumor target cells (Figure 5). 

Co-culture experiments demonstrated a boost of antitumoral effects. Viable tumor cell numbers notably decreased after a 48 hours incubation period especially at higher CT concentrations. Generally, antitumoral effects were rather cell specific, than donor dependent. Obvious tumor killing was found for T3M4 and BxPC-3 cells, while MIA PaCa-2 and AsPC-1 cells responded less. 

### 3.6. Delayed Tumor Growth following Local Treatment

Finally, an *in vivo* experiment was carried out to test whether the observed *in vitro* findings on potent tumor growth inhibition and immune stimulation are reproducible in immunocompetent, tumor-carrying mice. Treatment of established Panc02 tumors was attempted by repetitive CT application. Two treatment protocols were employed, including local as well as systemic application routes. 

**Figure 6:**
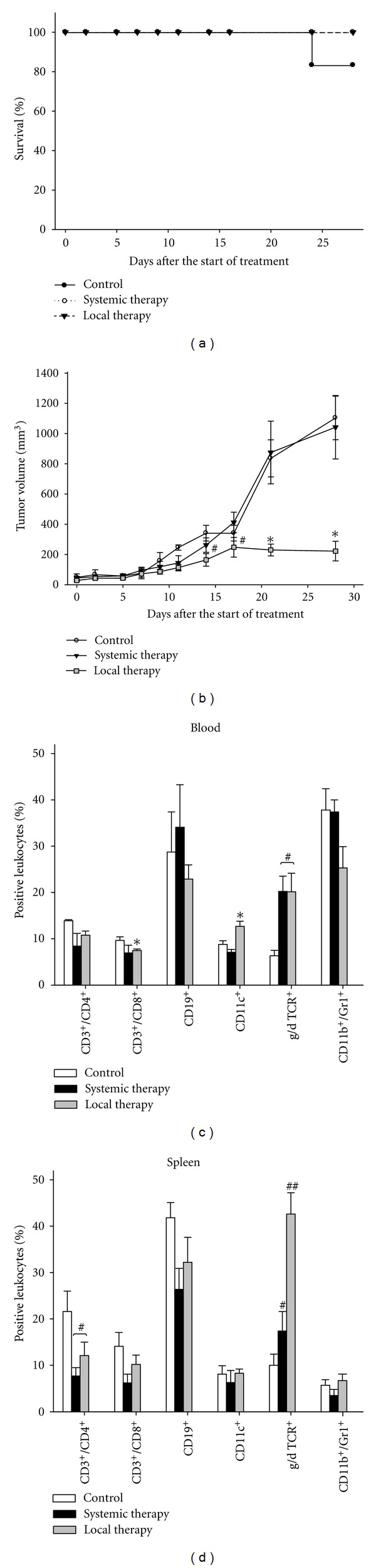
*In vivo *analysis. (a) Survival curve of Panc02 tumor-carrying C57Bl/6N mice and (b) tumor growth kinetic. After tumor establishment, mice received twice weekly i.t. or i.v. CT injections (six times in total (*n* = 3–7 mice per group). Control mice were given PBS. Mice were monitored for 28 days with adherence to ethical requirements. (c), (d) Flow cytometric phenotyping of circulating (blood) and splenic leukocyte subsets from tumor control and CT-treated animals. Values are given as mean ± SEM; *P < 0.05 versus control;* t*-test. ^#^ *P* < 0.05 versus control; *U*-test.

Both regimens were well tolerated by all animals. No signs of tumor-associated clinical symptoms like anorexia or weight loss occurred, consequently resulting in a 100% survival rate (Figure 6(a)). Local CT application mediated substantial tumor growth alteration. This was evident from day 14 after treatment start until the end of experiments (day 28). Established Panc02 tumors macroscopically necrotized, tended to break up, and revealed significant growth delay. Specifically, at day 28, tumor volumes were about one-fifth of control tumors (222.6 ± 65.0 mm³ versus 1103.8 ± 144.4 mm³, *P* < 0.05 versus control). By comparison, an increase in the number of injections did not further improve the therapeutic effect and thus resulted in equivalent tumor volumes (data not shown). 

Quite the opposite results were obtained following systemic CT application. This regimen had no impact on Panc02 tumor growth. Tumors continuously grew and volumes were similar to controls until terminating the experiment (Figure 6(b)). Therefore, local application is more favorable than systemic.

### 3.7. Induction of Immune Cells following Local Therapy

Next, the composition of circulating as well as splenic immune cells was studied (Figures 5(c) and 5(d)). In these analyses, *in vivo* findings could clearly be correlated with immunological parameters. Mice locally treated with CT had significantly increased numbers of circulating CD11c^+^ dendritic and *γ*/*δ* TCR^+^ cells (*P* < 0.05 versus control). Moreover, levels of myeloid derived suppressor cells (MDSC, CD11b^+^Gr1^+^) decreased exclusively after local CT therapy (Figure 6(c)). Irrespective of the application route, T helper and cytotoxic T cells as well as CD19^+^ pre B cells were only slightly altered. 

In striking contrast, locally as well as systemically applied CT impacted splenic T and B cells (Figure 6(d)). Both treatment groups had decreased numbers of these cell populations. This dramatic decrease at day 28 possibly reflects migration of immunological effector cells more into lymphoid tissues than into the spleen. Numbers of *γ*/*δ* TCR^+^ cells were significantly raised, especially in the local therapy group (local 42.6 ± 4.6% versus systemic; 17.4 ± 4.2% versus control 10.0 ± 2.4%). Expression levels of L-selectin (CD62L) on lymphocytes and transferrin-receptor-positive (CD71) cells remained comparable to controls (data not shown).

Taken together, these findings confirm our *in vitro* results on potent stimulation of immune responses by CT. However, they also prove dependency on the application route finally contributing to efficient tumor growth control *in vivo*. 

## 4. Discussion

The main objective of this study was to ascertain whether a purely microbial-based approach, in the absence of additional (chemo- or targeted) therapies, can cure experimental non-immunogenic tumors. We therefore picked up the historical idea of using Coley's Toxin (CT), a complex mixture of gram-positive and gram-negative bacterial components, as an active antineoplastic agent. Our work focused on pancreatic carcinomas since this tumor entity is still one of the most malignant ones with high mortality and poor prognosis even with aggressive conventional and novel combinatorial therapies [14, 15]. Besides, successful eradication of experimental pancreatic carcinomas by local application of vital as well as avitalized gram-positive bacteria was demonstrated by our group before [16, 17]. 

For potential clinical application of microbial-based vaccines, several requirements need to be complied. These include (I) reducing unspecific toxicity to normal, nonneoplastic cells, (II) preserving antitumoral and tolerance-breaking immunostimulatory potential, and (III) applying a standardized treatment protocol. As for the latter, no such standardization was done in the past. Hence, despite Coley's high profile, his work came under criticism. At that time, 13 different preparations and various administration routes (i.v., i.m., and i.t.) existed and some of these were more effective than others [2, 18]. This may explain why Coley's results could not be reproduced by others. In order to overcome this obstacle, we designed CT under constant, standardized conditions according to the original protocol. 

Results of our present study show that this preparation acts as a potent antitumoral and immune-activating agent. First, by performing a series of *in vitro* experiments, we were able to induce cell death in tumor target cells. Interestingly, a differential susceptibility towards CT was demonstrated. While AsPC-1 cells responded with substantial cell death, other cell lines were less affected. As central mechanisms of CT-induced growth alteration, we could identify an up-regulation of p21^waf^ gene expression and loss of G2/M phases, both indicative for cell cycle arrest. In line with the established capacity of bacteria to induce apoptosis as well as necrosis in target cells, we also observed both kinds of cell death. Proteins (i.e., LPS, Flagellin) delivered by *S. marcescens* may thus have preferentially induced necrosis, while factors provided by *S. pyogenes* (i.e., streptokinase, streptolysin, and lipoteichoic acid) led to apoptosis [1, 16, 19, 20]. Accordingly, caspase 3/7 activation and DNA fragmentation were detectable in CT-treated tumor cells. 

In addition to the capacity of directly compromising tumor viability, CT was described as being a strong immune stimulator [2, 21–23]. The bacterial DNA (CpG ODN) present in this complex mixture may here be one of the best known immune-activating candidates. CpG ODNs has been found to improve antigen-presenting cell functions and boost humoral as well as cellular Th1-directed immune responses. They have shown promising results as adjuvants for vaccines and in combination with radio- or immunotherapy [3, 5, 24]. Several CpG ODN-based agents were already included into clinical trials for exploring their safety and efficacy in hematological and solid cancers [24–26] and [http://www.clinicaltrials.org/]. The underlying mechanism is due to activating TLRs, the most important innate immune receptors [5, 7]. TLR signaling in immune cells is crucial for regulating innate and adaptive immune responses, such as DC maturation and antigen presentation as well as CD8^+^ T-cell toxicity [27, 28]. 

In our experimental setup, CT-stimulated leukocytes from healthy donors could be effectively activated and responded with up-regulation of TLR 2, 5, and 9. Likewise, CD25 expression was significantly and sustainably induced in these short-time-mixed leukocyte cultures. Though not analyzed in detail, stimulation of *γ*/*δ* T cells is very likely [29]. Besides, secretion of Th1 and other proinflammatory cytokines (e.g., IFN-*γ*, IL12, and TNF-*α*) by immune cells belonging to both the innate and adaptive arm can be anticipated, too. Hence, this mixture of TLR agonists likely stimulates a complex cascade, each of which plays a unique and vital role in orchestrating immune responses [30]. Quite in line, we observed a boost of antitumoral effects when leukocytes were added to tumor cells together with CT. This co-culture system mimics the *in vivo* situation. Here, tumor cell numbers massively decreased. Worth mentioning, however, is the fact that boosted antitumoral effects were rather tumor cell specific than dependent from the lymphocyte donor. T3M4 and BxPC-3 could be effectively killed by CT and leukocytes. However, comparable results were not obtained for AsPC-1 cells, which had been shown to be highly susceptible towards CT-mediated lysis alone. This can be attributed to a kind of tumor-escape mechanism. These cells probably secrete immunosuppressive factors (IL10, TGF-*β*), thereby preventing leukocyte stimulation and immune-mediated lysis. 

In a subsequent syngeneic *in vivo* tumor model, CTs' potential to impact solid tumors was examined. Again, we focussed on a non-immunogenic tumor, although it is clearly established that low immunogenic tumors respond worse than their immunogenic counterpart [2]. Nonetheless, we observed strong oncopathic effects following local, repetitive administration in immune-competent mice bearing syngeneic Panc02 pancreatic carcinomas. This finally resulted in significantly delayed tumor growth. Of particular interest was the finding that maximal tumor growth control was obtained after six injections. Increasing the number of injections did not further boost therapeutic responses. Hence, CT may thus be best combined with other (antineoplastic) drugs rather than used as a single agent. In the case of pancreatic carcinomas, this would probably include gemcitabine, since this drug is still the most effective one [31, 32]. However, before further exploring such combinatorial approaches possible intolerable toxic side effects (e.g., cardiac, gastrointestinal, and hematological toxicity, anorexia, neuropathy, arthralgia, and myalgia) have to be excluded or at least minimized. Additional to identifying the optimal nontoxic dose, a proper application route (i.e., systemic versus local), an appropriate and feasible time schedule (simultaneous versus consecutive therapy), and potential synergistic or antagonistic effects of selected combinations have to be evaluated. In our experiments, we even observed differential responses using different application routes. Systemic applications failed to affect Panc02 tumors. This experimental observation fits to the historical fact of Coley himself primarily treating patients with soft tissue sarcomas. Those can be easily accessed and this might finally explain Coley's impressive clinical successes [2, 33]. 

In line with Coley's intuitions of stimulating the immune system to be effective in treating cancer, therapeutic effects following local CT application could be attributed to systemic immune activation primarily belonging to the innate arm. These included significantly increased numbers of circulating DCs and slightly reduced MDSC levels. MDSCs are by definition immature and contribute to tumor-related immunosuppression by inducing T- and NK-cell dysfunction [34]. Only recently, Zoglmeier and Coworkers described that several TLR-Ligands (e.g., CpG, Poly I:C) block the suppressive function of MDSCs by inducing maturation and differentiation, however, without affecting their total numbers [35]. These findings are in agreement with our observations on only marginal reductions in total circulating and splenic MDSCs levels, but efficient tumor growth control *in vivo*. We therefore hypothesize that local CT therapy was able to convert MDSC functions that allowed for immune-mediated tumor growth inhibition. Gaining deeper insights into the underlying mechanisms is of particular interest for subsequent follow-up studies.

Somewhat unexpected and yet partly unexplained remains the massive increase in *γ*/*δ* TCR^+^ cells, especially in spleens of locally treated mice. These cells provide a link between innate and specific immune responses, capable of mediating non-MHC-restricted tumor lysis [36]. Additionally, they were found to act against invading pathogens by producing IFN-*γ* [37, 38]. In this regard, increased numbers of *γ*/*δ* TCR^+^ cells may here be best interpreted as a mixture of both functions, that is, inducing Panc02 tumor lysis and acting against invading bacterial components in order to mediate host defense. 

Taken together, we have shown that CT is still worth being employed for cancer immunotherapy due to its direct antitumoral as well as indirect immunostimulatory capacity. Accordingly, some standardized commercial bacterial extracts (MBVax, Vaccineurin, OK-432) comparable to the original CT exist nowadays. However, they mostly failed to prove effective in the clinics [9, 39]. Firstly, high fever, frequently occurring following infusion, was designated as being a stop criterion. Secondly, treatments were usually short lived, and thirdly, most of the patients were immunocompromised at time of treatment due to earlier or concurrent chemotherapy. Hence, before translating such microbial-based vaccines into clinical trials, carefully deliberating in- and exclusion criteria as well as treatment schedules is crucial. 

## 5. Conclusions

In the era of increasing cancer incidence, the use of microbial vaccines for immunotherapy is still being re-examined. In this regard, the historical bacterial mixture of Coley's Toxin has here been shown of exhibiting antitumoral and immunostimulatory potential *in vitro* as well as *in vivo*. Data presented herein prove that this approach may provide a basis for local repetitive administration into tumor-carrying hosts, which would be best applied as combinatorial agent. Hence, Coley's Toxin still holds promise for further preclinical analyses that could finally contribute to a successful treatment regimen *in vivo*. 

## Figures and Tables

**Table 1:** Cell cycle distribution of tumor control cells and following CT treatment.

	MIA PaCa-2				T3M4				BxPc-3				AsPC-1			
Phase	0	0.05 ∗ 10^4^	0.25 ∗ 10^4^	25 ∗ 10^5^	0	0.05 ∗ 10^4^	0.25 ∗ 10^4^	25 ∗ 10^5^	0	0.05 ∗ 10^4^	0.25 ∗ 10^4^	25 ∗ 10^5^	0	0.05 ∗ 10^4^	0.25 ∗ 10^4^	25 ∗ 10^5^
	cfu/mL				cfu/mL				cfu/mL				cfu/mL			
G0/G1	48.7 ± 9.4	48.6 ± 13.9	43.1 ± 13.0	43.6 ± 0.7	77.5 ± 1.1	76.7 ± 3.5	67.4 ± 3.8	64.5 ± 2.2	44.7 ± 8.5	43.0 ± 1.0	31.8 ± 4.1	36.6 ± 9.9	48.1 ± 10.1	52.7 ± 6.8	37.5 ± 8.9	30.7 ± 3.6

S	33.4 ± 9.5	36.5 ± 10.4	43.1 ± 12.7	50.5 ± 6.2	18.0 ± 0.9	18.2 ± 4.5	28.7 ± 5.1	31.1 ± 1.5	24.1 ± 5.5	44.8 ± 7.1	68.0 ± 4.4*	63.1 ± 7.9*	39.5 ± 11.3	34.3 ± 11.5	58.9 ± 9.7	67.9 ± 3.1^#^

G2/M	17.9 ± 3.9	15.6 ± 3.4	13.8 ± 0.9	8.5 ± 5.5	4.6 ± 0.3	5.1 ± 1.2	4.0 ± 1.3	4.4 ± 0.7	31.2 ± 3.2	8.8 ± 5.0*	0.4 ± 0.4*	0.3 ± 0.2^#^	16.7 ± 5.9	13.0 ± 5.3	0.4 ± 0.4^#^	0.4 ± 0.1^#^

Data show percentage values of tumor cells (% positive cells) following 24 hours of treatment. Experiments were at least three times repeated. Values of are given as mean ± SEM; *P < 0.05 versus control;* t*-test. ^#^ *P* < 0.05 versus control; *U*-test.

**Table:** (a)

Antigen	Donor 1				Donor 2				Donor 3				Donor 4			
	0	0.05 ∗ 10^4^	0.25 ∗ 10^4^	25 ∗ 10^5^	0	0.05 ∗ 10^4^	0.25 ∗ 10^4^	25 ∗ 10^5^	0	0.05 ∗ 10^4^	0.25 ∗ 10^4^	25 ∗ 10^5^	0	0.05 ∗ 10^4^	0.25 ∗ 10^4^	25 ∗ 10^5^
	cfu/mL				cfu/mL				cfu/mL				cfu/mL			
CD3^+^CD4^+^	35.4	34.9	38.3	45.3	26.7	24.9	24.5	37.9	40.8	41.2	31.6	49.3	43.9	31.0	35.6	51.6
CD3^+^CD8^+^	17.8	18.9	19.5	22.3	13.7	15.9	14.7	16.8	20.8	21.6	18.5	24.2	9.5	10.8	9.9	12.2
CD25^+^	22.2	23.4	39.2	46.6	12.3	18.7	29.2	41.1	12.4	12.9	20.1	29.6	10.0	11.5	22.8	33.8
CD16^+^CD56^+^	14.2	17.0	14.8	17.1	30.1	23.7	23.7	22.2	11.1	9.7	10.8	9.7	12.2	10.3	11.3	10.4

Data show percentage values of huPBLs (% positive cells) following 24 hours of stimulation.

**Table:** (b)

Antigen	Donor 1				Donor 2				Donor 3				Donor 4			
	0	0.05 ∗ 10^4^	0.25 ∗ 10^4^	25 ∗ 10^5^	0	0.05 ∗ 10^4^	0.25 ∗ 10^4^	25 ∗ 10^5^	0	0.05 ∗ 10^4^	0.25 ∗ 10^4^	25 ∗ 10^5^	0	0.05 ∗ 10^4^	0.25 ∗ 10^4^	25 ∗ 10^5^
	cfu/mL				cfu/mL				cfu/mL				cfu/mL			
CD3^+^CD4^+^	33.4	38.2	42.6	52.5	16.0	16.2	22.0	33.2	31.0	33.6	31.7	53.7	35.5	35.0	33.5	61.0
CD3^+^CD8^+^	14.3	13.8	18.5	20.8	11.2	13.0	12.5	15.1	20.3	22.2	22.4	26.1	10.2	8.9	11.8	13.1
CD25^+^	9.0	18.5	23.04	28.8	11.0	20.5	25.4	33.3	9.8	20.8	21.4	25.6	9.7	20.5	15.9	28.9
CD16^+^CD56^+^	8.9	8.1	8.0	8.0	16.5	27.6	14.2	14.5	5.8	8.4	6.4	6.2	7.8	2.5	6.5	7.8

Data show percentage values of huPBLs (% positive cells) following 72 hours of stimulation.

## Abbreviations

LPS: Lipoteichoic acid
CT: Coley's Toxin
Cfu: Colony forming unit
DC: Dendritic cell
MDSC: Myeloid derived suppressor cells
TLR: Toll-like receptor
huPBL: Human peripheral blood leukocytes
PI: Propidium iodide.
